# Medial Temporal Lobe Involvement in Human Prion Diseases: Implications for the Study of Focal Non Prion Neurodegenerative Pathology

**DOI:** 10.3390/biom11030413

**Published:** 2021-03-10

**Authors:** Alberto Rábano, Carmen Guerrero Márquez, Ramón A. Juste, María V. Geijo, Miguel Calero

**Affiliations:** 1 Neuropathology Department, Alzheimer’s Disease Research Unit, CIEN Foundation, Institute of Health Carlos III, Queen Sofía Foundation Alzheimer Research Center, 28031 Madrid, Spain; 2 CIEN Foundation and Centro de Investigación Biomédica en Red sobre Enfermedades Neurodegenerativas (CIBERNED), Institute of Health Carlos III, 28031 Madrid, Spain; mcalero@isciii.es; 3 Neurological Tissue Bank—HUFA Biobank, Hospital Universitario Fundación Alcorcón, 28922 Madrid, Spain; cguerrero@fhalcorcon.es; 4 Department of Animal Health, NEIKER-Basque Institute for Agricultural Research and Development, Basque Research and Technology Alliance (BRTA), Parque Científico y Tecnológico de Bizkaia P812, 48160 Derio, Spain; rjuste@neiker.eus (R.A.J.); mgeijo@neiker.eus (M.V.G.); 5 Chronic Disease Program, Institute of Health Carlos III, 28222 Madrid, Spain

**Keywords:** human prion diseases, medial temporal lobe, neurodegenerative diseases, prion strains

## Abstract

Human prion and non-prion neurodegenerative diseases share pathogenic mechanisms and neuropathological features. The lesion profile of a particular entity results from specific involvement of vulnerable neuron populations and connectivity circuits by a pathogenic protein isoform with strain-like properties. The lesion profile of the medial temporal lobe (MTL) was studied in postmortem tissue of 143 patients with human prion disease (HPD) including sporadic, genetic, and acquired forms. Most cases (90%) were classified according to PrP^res^ type and/or *PRNP* codon 129 status, in addition to a full neuropathological profile. Mixed histotypes represented 29.4% of total sporadic Creutzfeldt-Jakob disease (sCJD) cases. An intensity score of involvement including spongiosis and astrogliosis was determined for the amygdala, presubiculum, subiculum, entorhinal cortex, CA1 to CA4 sectors of the hippocampal cortex, and dentate gyrus. Connectivity hubs within the MTL presented the highest scores. Diverse lesion profiles were obtained for different types and subtypes of HPD. Impact of mixed PrP^res^ types on the MTL lesion profile was higher for sCJDMV2K cases than in other histotypes. Differences between MTL profiles was globally consistent with current evidence on specific strains in HPD. These results may be relevant for the analysis of possible strain effects in focal non-prion neurodegenerative conditions limited to the MTL.

## 1. Introduction

It is widely accepted that non prion neurodegenerative entities, like Alzheimer’s, Parkinson’s, or Huntington’s disease, share multiple features at a molecular, pathogenic, and neuropathological level with what we can still properly call human prion diseases (HPD) stricto sensu. Terms like “prion-like” and “prionoid” are commonly used to describe these singular features (including conformational strain-like properties and experimental cell-to-cell spreading of pathogenic protein isoforms) observed, for example, in tauopathies, synucleinopathies, and TDP-43 pathologies, among others [1,2,3]. However, despite compelling evidence of iatrogenic person-to-person transmission of Alzheimer’s type amyloid-β pathology [4,5], there is still a wide gap in terms of pathogenic mechanisms between prionopathies and other neurodegenerative proteinopathies. HPD display, in a rather sharp way, phenotypic and pathogenic features that in other neurodegenerative diseases are somewhat blurred by associated factors of complexity. For all etiologic forms of HPD (sporadic, genetic or acquired prionopathies), a genetic polymorphism is a major determinant of genetic risk of disease. Additionally, certain molecular characteristics of the pathologic prion protein (PrP^Sc^) (“strain” features) are regularly associated with specific neuropathological and clinical disease phenotypes [6,7]. Accordingly, HPD may represent particularly useful models for exploring two related factors that determine phenotypic variability in all neurodegenerative disorders: (i) differential vulnerability of specific cellular (both neuronal and glial) populations to pathogenic factors [8], and (ii) involvement of specific neural pathways that may facilitate the spread of pathology throughout the central nervous system [9].

The medial temporal lobe (MTL) is one of the major brain regions involved in neurodegenerative pathology in human beings. MTL structures include the amygdaloid complex (amygdala), the hippocampal formation, entorhinal cortex, and neighbor cortical areas (transentorhinal and perirhinal cortices) [10]. Both intrinsic and extrinsic connectivity of the MTL is rather well-known in the human brain. High connectivity of some nuclei of the amygdala, the entorhinal cortex, and hippocampus to extratemporal cortical regions and subcortical nuclei may contribute to extensive spread of pathology beyond the MTL in diffuse neurodegenerative conditions like Alzheimer’s disease and Lewy body dementia [11,12]. Moreover, in early stages of disease progression (e.g., Braak stages I to III for Alzheimer’s type neurofibrillary pathology), the topographic pattern of involvement is consistent with local connectivity within the MTL [13].

Recently, several new entities have been added to the group of focal neurodegenerative conditions that either progress within the limits of the MTL or show a predominantly MTL distribution of pathology. In both cases, the starting point of the disease is assumed to be located within the MTL or in a closely related region. This group comprises a synucleinopathy (Lewy body disease limited to or predominant in the amygdala [LBD-A]) [14], several tauopathies (argyrophilic grain disease [AGD] [15], primary age-related tauopathy [PART] [16], aging-related tau astrogliopathy [ARTAG]) [17], and one disorder associated with TDP-43 pathology (limbic-predominant age-related TDP-43 encephalopathy [LATE]) [18]. All these conditions are mostly associated with advanced age and develop commonly in combination with other neurodegenerative or non-neurodegenerative (mainly cerebrovascular) diseases. What prevents extensive extratemporal propagation of pathology in these focal disorders, in contrast with their massively diffuse counterparts (Alzheimer’s disease, Lewy body disease, diffuse tauopathies like progressive supranuclear palsy, or frontotemporal lobar degeneration-TDP) is currently unknown. However, they also seem to display a topographical distribution of histological lesions that is consistent with the intrinsic circuitry of the MTL. Limitation of pathology to the MTL in these focal entities may be codified in the strain properties of the corresponding pathologic protein isoforms.

In contrast, HPD are characteristically diffuse, neurodegenerative disorders that affect, mostly through a rapidly progressive evolution, wide and distant regions of the central nervous system. As above-mentioned, the phenotypic profile of each HPD entity is highly dependent on the strain-like characteristics of the specific associated PrP^Sc^ isoform. This is particularly manifest in sporadic Creutzfeldt–Jakob disease (sCJD), where the combination of the PrP^res^ type present in brain tissue and the patient’s haplotype of *PRNP* gene codon 129 largely determine the neuropathological and clinical diversity of cases [19]. A comparably close relationship between PrP^Sc^ molecular features and disease phenotypes has also been observed in other sporadic HPD (e.g., variably protease-sensitive prionopathy [VPSPr]) as well as in genetic and acquired prion disorders [7].

Involvement of the MTL is also frequent, though highly variable, in HPD, and seems to represent a characteristic phenotypic feature of some prionopathies. The aim of the present study is to describe the lesion profile observed in the main anatomical structures of the MTL in a wide variety of HPD, and to analyze possible associations between specific patterns of involvement and PrP^Sc^ strains related to different prionopathies. A similar approach may be applied to MTL involvement by other neurodegenerative conditions, particularly those focal disorders that remain limited to the MTL or show predominant involvement of this key neuroanatomical region.

## 2. Materials and Methods

### 2.1. Neuropathological Procedures

This study was based on the neuropathological postmortem examination of patients with clinically suspected HPD and diagnosed at the Hospital Universitario Fundación Alcorcón (HUFA) in Madrid, Spain, a national reference center for postmortem diagnosis of HPD. Patients included in the study corresponded to all neuropathological autopsies performed consecutively between 1999 and 2009. The Spanish National CJD Surveillance System has established several reference centers for postmortem diagnostic confirmation of cases throughout the country [20]. Some regions (e.g., Catalonia, Basque Country, Galicia, and Andalusia) run their own local reference centers. Accordingly, HUFA receives patients for neuropathological autopsy mainly from the Madrid region and from other regions lacking a local reference center (i.e., both Castilles (Castilla—La Mancha and Castilla y León), Comunidad Valenciana, Murcia, Extremadura, Aragón and the Balearic and Canary Islands).

Postmortem interval (PMI) varied between 6 and 24 h. The cadaver was maintained at 4 °C for long PMIs before autopsy. Brain extraction was performed under strict biosafety measures, and several fresh tissue samples (right frontal and parietal lobes, and right cerebellar hemisphere) were obtained for freezing and long-term preservation at −80 °C. The rest of the brain tissue was fixed by immersion in 4% buffered formaldehyde. After 3–4 weeks of fixation, the brain was cut and sampled for paraffin embedding and further histological processing. The cerebral hemispheres were serially cut in coronal slices, whereas the cerebellum was sliced in the sagittal plane and the brainstem in a transversal plane (perpendicular to Meynert’s axis). Around 25 tissue blocks were obtained from each brain including cortical and subcortical brain regions and the MTL was sampled in three coronal planes at the level of the amygdala, anterior (head), and posterior (body) hippocampus, respectively. The anatomical limit between the anterior and posterior hippocampus was defined by the tip of the uncus. Cerebral hemispheric tissue blocks for neuropathological diagnosis including MTL samples were obtained from the left hemisphere in all cases. Before further paraffin embedding, fixed tissue blocks were decontaminated by immersion in 98% formic acid.

Histological assessment of cases was performed through routine hematoxylin eosin (H/E) staining of sections (5 μm thickness) from sampled tissue blocks, and immunohistochemical staining for PrP of selected brain regions (frontal cortex, striatum, and cerebellar cortex). A Nikon Eclipse 90i microscope with apochromatic optics was employed for histological evaluation. Monoclonal antibody 3F4 (Sigma, St. Louis, MO, USA) was used as a primary antibody for immunohistochemistry. Definite neuropathological diagnosis of HPD was carried out according to widely accepted morphological criteria [21] based on the topographical distribution of gliosis and neuronal loss, and on the pattern of spongiosis (H/E) and protease-resistant prion protein (PrP^res^) deposits in immunohistochemistry (frontal cortex, striatum, and cerebellum). For the diagnosis of iatrogenic CJD (iCJD), a history of medical/surgical exposure was considered, and genetic prionopathies were diagnosed on the basis of evidence of specific pathogenic mutations in *PRNP*. Final classification of sCJD cases included data of *PRNP* codon 129 haplotype (Section 2.2) and of PrP^res^ molecular typing (Section 2.3).

### 2.2. Genotyping

Total DNA was isolated from peripheral blood. The analysis of potential mutations and the polymorphism at codon 129 (rs1799990) in the *PRNP* gene was performed by DNA sequencing as described [22]. For each patient, codon 129 status was established as methionine/methionine (MM), methionine/valine (MV), or valine/valine (VV).

### 2.3. Molecular Typing of PrP^res^

Western blot analysis was performed by standard methods [23,24]. Specifically, 1.5 mL of lysis buffer with zirconium beads were added to 0.15 g of brain in screw cap 2.5 mL tubes. These were submitted to 45 s shaking in a Precellys homogenizer. Then, the tubes were centrifuged for 5 min in a Haereus microfuge at 4 °C. The supernatant was transferred to a 1.5 mL Eppendorf tube and frozen at −20 °C until use. After thawing at 4 °C, a 197 μL aliquot was transferred to a 1.5 mL Eppendorf tube where 2.5 μL of proteinase K (8 mg/mL, for a final 100 μg/mL concentration) were added and incubated at 37 °C for 1 h. The reaction was stopped with 10 μL Pefabloc 20 nM (1 mM final concentration). A 30 μL aliquot was transferred to 96-well plates and stored until use. An equal volume of 2× loading buffer (modified from 1: 125 mM Tris-HCL pH 7; 4% sodium dodecyl sulpahte solution (SDS); 20% glycerol; 0.02% bromophenol blue; 200 mM dithiothreitol [DTT]) was added and the samples were denatured at 96°C for 8 min before electrophoresis on 16% SDS-Tris-glycine gels (5% stacking) for 90 min at 150 V. The Precision Plus protein Unstained Standards Molecular weight marker was used. The gels were electroblotted onto a Polyvinylidene difluoride (PVDF) membrane (Immobilon-P, Millipore, Burlington, VT, USA) and blocked as described elsewhere [24]. After a short wash in PBST 1×, membranes were incubated with anti-PrP monoclonal antibody 3F4 (epitope 109–112: MKHM) (Sigma, St. Louis, MO, USA) (1:3.000) for 1 h. Following a washing step in PBST 1× for 45 min, membranes were incubated with an alkaline phosphatase conjugated goat anti-mouse IgG antibody (Sigma, St. Louis, MO, USA) diluted 1:10.000 for 1 h at room temperature or at 4 °C overnight. After a wash step of 45 min in PBST 1× and equilibration in 200 mM Tris-HCl; 10 mM MgCl_2_, pH 9.8 2 for 5 min, membranes were developed in a chemiluminescent substrate (Immun-Star Substrate, Bio-Rad, Hercules, CA, USA) and visualized on Amersham Hyperfilm ECL. The plates were scanned and stored for image processing and molecular weight and intensity calculation. Each sample PrP was typed according to Parchi et al. (1997) [25].

### 2.4. Case Series and Classification

A total of 143 HPD patients were included in the study, classified either as sporadic CJD (*n* = 126), genetic HPD (*n* = 12 including eight familial CJD and four fatal familial insomnia cases [FFI]), and acquired CJD (*n* = 5 including two iatrogenic CJD and three variant CJD cases). Full molecular classification (codon 129 status and PrP^res^ type present in the cerebellum) was available for 98 cases (68%), whereas in 32 cases (22%), only genetic data were available. For 10% of cases, final classification was based only on neuropathological information. The distribution of cases between diagnostic levels (full molecular vs. only genetic vs. only neuropathological) was equivalent between HPD types and subtypes (particularly for sCJD histotypes). In 81 cases (83% of cases with PrP^res^ typing of the cerebellar tissue sample), an additional PrP^res^ typing was available for the frontal lobe sample. Among sporadic CJD cases, 42 cases (29.4%) showed a mixed molecular subtype, based on the identification of PrP^res^ types 1 and 2a in a single brain tissue sample or in two different samples from the same patient. These cases were classified as mixed sCJD cases [26]. Some atypical features were observed in a small group of sCJD cases. A panencephalopatic phenotype was observed in seven cases (six MM and one MV), all of them showing PrP^res^ type 1 in frontal lobe and cerebellum samples. In eight additional sCJD MM1 cases, some VV2-like features were observed in the hippocampus, while 2 sCJD VV2 cases displayed histological lesions suggestive of sCJD VV (2 + 1). No MM2-thalamic type (MM2T) case was identified in the patient series. All familial CJD (fCJD) cases were associated with the E200K mutation, and all of them, except one (MV), were associated with the MM haplotype at codon 129. In all cases with molecular typing of PrP^res^ available, type 1 PrP^res^ was detected in both brain tissue samples. One of the FFI cases, bearing the MV genotype, presented a fCJD-like neuropathological phenotype. One of our iCJD was associated with previous neurosurgery, while the second case was due to a dura matter graft implant. Both iCJD presented a MM status at codon 129 and type 1 PrP^res^ in brain tissue. Table 1 shows the distribution of cases between HPD diagnostic categories.

### 2.5. Anatomical Regions and Morphological Variables

**Figure 1:**
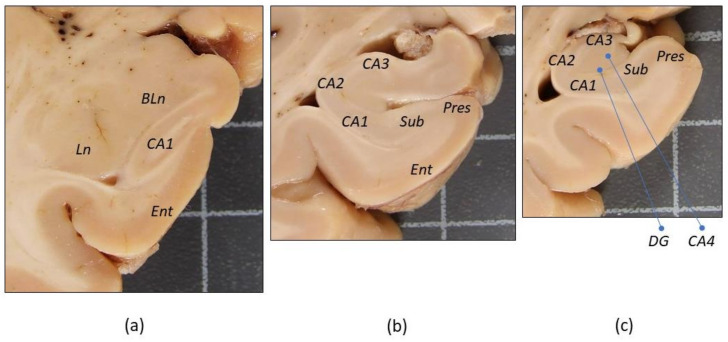
Macroscopic images of medial temporal lobe coronal sections showing the main anterior–posterior levels and regions of interest assessed in the present study. Photographs correspond to a control brain. (**a**) Amygdala, (**b**) anterior hippocampus (head), and (**c**) posterior hippocampus (body). The location of assessed areas of interest is indicated at each level. *BLn*, basolateral nuclei, and *Ln*, lateral nuclei of the amygdala. *Ent*, entorhinal cortex. *Pres*, presubiculum. *Sub*, subiculum. *CA1*, *CA2*, *CA3* and *CA4*, Sommer’s sectors of the hippocampus cortex. *DG*, dentate gyrus. Squares of the background grid are 1 cm in length.

For the histological evaluation of the MTL, three coronal levels were selected in an anterior–posterior sequence: amygdala (Am), anterior hippocampus (Ha), and posterior hippocampus (Hp) (Figure 1). The following areas of interest were assessed at either one or two of these coronal levels (in brackets): amygdala (Am), entorhinal cortex (Ha), presubiculum (Hp), subiculum (Hp), CA1 (Hp and Ha), CA2, CA3, and CA4 sectors of the hippocampal cortex (Hp), and dentate gyrus (Hp). Morphological variables were registered for the CA1 sector at both the anterior and posterior hippocampus level. For each area of interest, the following morphological variables were assessed in H/E stained histological sections:

Spongiosis, intensity (Si), scored from 0 to 4. For CA1–3 sectors of the hippocampus, intensity of spongiosis was registered separately for the pyramidal layer (stratum pyramidale) and for the superficial layers of the cortex (stratum lacunosum-moleculare, stratum radiatum, and stratum lucidum).
Vacuolation (type): only small vacuoles (1), only medium size to large vacuoles (2), predominantly small vacuoles (3), predominantly medium size to large vacuoles (4), minimal vacuolation (5).
Astrogliosis, intensity (Ag), scored from 0 to 4.
Average intensity of involvement (Ai): Si + Ag/2. For CA sectors of the hippocampus, Si score for the pyramidal layer was selected.
Ballooned neurons (frequency), scored from 0 to 3. This variable was only assessed in amygdala and entorhinal cortex sections.
Laminar spongiosis: presence of laminar spongiosis, and cortical layers involved by spongiosis with a laminar distribution (assessment limited to the entorhinal cortex).

### 2.6. Statistics

Statistical analysis and graphs were performed with the aid of SPSS 24.0 software. Due to the diverse and generally low number of patients per study group, and to non-normal distribution of the main outcome variables, non-parametric statistical tests were employed for bivariate analysis: Mann–Whitney U test for comparison of two groups, Kruskal–Wallis test for comparison of more than two groups, and Spearman’s test for analysis of correlation between variables.

### 2.7. Ethical and Legal Issues

All neuropathological autopsies and diagnostic procedures were performed within the framework of the Spanish National CJD Surveillance System. Biological samples and associated data were handled in compliance with national regulations concerning clinical autopsy practice and biomedical research including informed consent by a proxy and anonymization of personal data. This study was approved by the local Ethics Research Board of the Hospital Universitario Fundación Alcorcón.

## 3. Results

### 3.1. Amygdala

Intensity of spongiosis (Si) and astrogliosis (Ag) was globally high in the case series, with significant correlation between both variables (Spearman test, *p* < 0.01). Average intensity of involvement (Ai) reached 1.79 (1.17) [mean (SD)] and showed significant variation between groups (Kruskal–Wallis test, *p* < 0.01). In particular, Ai was highest in sCJD VV2, MV2K, MV2K + C, VV1, and iCJD groups, and was significantly higher for sCJD VV2 compared to sCJD MM/MV1 (Mann–Whitney U test, *p* < 0.01) (Table 2). Whereas this latter group displayed a wide range of variability, with more intense and diffuse changes observed in cases of the panencephalopathic type, Ai was uniformly high in the sCJD VV2 group. No difference was observed for Ai between pure sCJD MM/MV1 and mixed sCJD MM/MV (1 + 2). The lowest Ai scores were observed in the FFI and sCJD MM/MV2 groups. The presence of ballooned neurons was relevant in sCJD VV2, and in a lesser degree, in sCJD MV2K + C. Low-grade involvement of the amygdala was limited to the basolateral nuclei, while more intense changes extended to other areas, particularly the lateral nucleus.

### 3.2. Entorhinal Cortex

As observed in the amygdala, Si and Ag scored high in the entorhinal cortex for the whole case series and showed significant correlation (*p* < 0.01) between them. However, while Si varied significantly with disease type and subtype, Ag did not. Average intensity (Ai) showed lower scores only in FFI, fCJD, and vCJD. No significant differences were observed in Ai between the largest groups, namely sCJD MM/MV1, MM/MV (1 + 2), and VV2. A high number of ballooned neurons was observed in sCJD VV2 and iCJD, and to a lesser extent among sCJD MV2K + C and MV2K + 1 cases. A laminar distribution of spongiosis was observed in a high number of cases (67.4%) in all diagnostic groups. Most frequently, layers II, III, and V of the entorhinal cortex were involved in the same case, while some cases displayed combined laminar spongiosis of layers II and III, III and V, or, less frequently, isolated involvement or layers II, III, or V.

### 3.3. Presubiculum and Subiculum

Average involvement (Ai) of the presubiculum was high in most diagnostic groups (2.69 [0.89]; mean [SD]), with mild gliosis. Only FFI and vCJD cases showed low scores at this level. In contrast, subiculum displayed a global low-grade, though highly variable average involvement, with particularly high scores among the sCJD VV2, MV2K, VV1, and MV2K + C groups. As observed in the amygdala, Ai differed significatively between the sCJD MM/MV1 and VV2 cases (Mann–Whitney U test, *p* < 0.01), though not compared to the sCJD MM/MV (1 + 2) group.

### 3.4. CA1 to CA4 Sectors of the Hippocampus

Spongiosis and astrogliosis were mild to moderate in CA1 in the case series considered globally, though high scores for average involvement (Ai) were observed in the sCJD VV2 and MV2K groups and, with a higher variability, in sCJD MV2K + C and MV2K + 1. As observed in other areas, differences between sCJD MM/MV1 and VV2 were significant, while no differences were observed between sCJD MM/MV1 and sCJD MM/MV (1 + 2). A high correlation was observed between spongiosis in the superficial cortical layers and spongiosis in the pyramidal layer (Spearman’s test, *p* < 0.01) of all CA sectors. However, in CA1, spongiosis limited to the superficial layers was higher and more extensively distributed between diagnostic groups, with the highest scores observed in sCJD VV2, MV2K, and MV2K + C. Average involvement score (Ai) at the CA1 sector showed high correlation between the anterior and posterior hippocampus, and no significant gradient effect was observed between both coronal levels.

**Figure 2:**
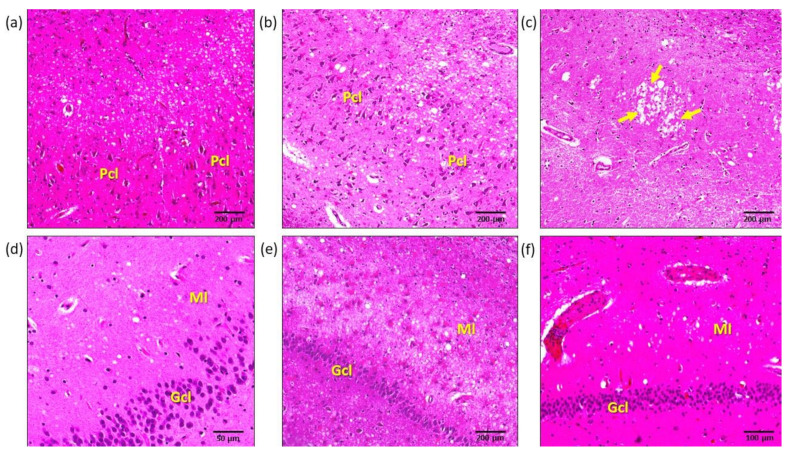
Microphotographs of characteristic histological lesions observed with hematoxylin-eosin stain in the hippocampus of cases included in this study. (**a**) CA2 hippocampus sector in a sCJD MM/MV1 case, showing mild spongiosis in the pyramidal cell layer (*stratum pyramidale*) and intense spongiosis in superficial layers. (**b**) CA2 sector in sCJD VV2 case, where intense spongiosis affects both the pyramidal cell layer and superficial layers. (**c**) *Lacunosum-moleculare* layer of the hippocampus at the CA2 level in a vCJD case, showing a characteristic pattern of spongiosis including a cluster of “florid plaques” (arrows). (**d**) Dentate gyrus of a sCJD MM/MV1 case, with minimal spongiosis in the molecular layer. (**e**) Intense dentate gyrus spongiosis and astrocytosis in a sCJD VV2 case. Note the presence of abundant hypertrophic astrocytes in all layers. (**f**) Minimal spongiosis in the dentate gyrus of a sCJD VV1 case. *Pcl*: pyramidal cell layer; *Gcl*: granular cell layer; *Ml*: molecular layer.

The CA2 sector displayed a pattern of involvement globally similar to CA1 for all variables (Figure 2a–c). The single difference observed at this level was a lower average involvement in sCJD MM/MV (1 + 2) cases compared to sCJD MM/MV1 (*p* < 0.05).

The CA3 hippocampal cortex displayed a pattern similar to those of CA1 and CA2, though with an even lower degree of intensity for all variables. Some degree of intensity for average involvement was observed in the sCJD VV2, MV2K, VV1, MV2K + C, MV 2K + 1, and fCJD groups.

In CA4, only sCJD VV2 reached moderate intensity of involvement, while a lesser degree was observed in sCJD MV2K, MM/MV 2C, VV1, MV2K + C, fCJD, and iCJD groups.

### 3.5. Dentate Gyrus

Dentate gyrus showed a wide variability of involvement, and most diagnostic groups displayed low intensity scores. Only sCJD VV2, MV2K, and MV2K + C reached moderate to high scores. As observed in CA2 to CA4, sCJD MM/MV1, and MM/MV (1 + 2) differed at this level due to the lower scores observed in the latter group (*p* < 0.05) (Figure 2d–f).

### 3.6. Vacuolation Pattern

For all disease groups, the type of vacuolation did not vary significantly between MTL assessed regions (Table 3). All disease types maintained a notably stable pattern along the different MTL areas, which was consistent with the global pattern present in other CNS regions. Notably, mixed MM/MV (1 + 2) cases displayed a higher variability of patterns than the MM/MV1 group in most anatomical areas, though maintaining the global predominant pattern present in pure cases.

### 3.7. Lesion Profiles

**Figure 3:**
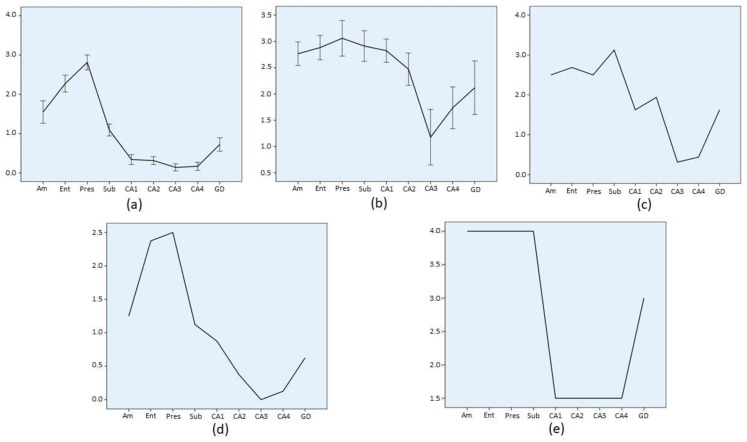
Regional profile of average involvement (Ai) intensity along the medial temporal lobe of sCJD histotype groups. Each group encompasses pure and mixed (PrP^res^ 1 + 2a) phenotypes. Confidence interval bars (95%) are shown only for groups with *n* > 30. Note the different scale range of individual charts. (**a**) sCJD MM/MV1, (**b**) sCJD VV2, (**c**) sCJD MV2K, (**d**) sCJD MM/MV2C, and (**e**) sCJD VV1. *Am*: amygdala; *Ent*: entorhinal cortex; *Pres*: presubiculum; *Sub*: subiculum; *CA1*, *CA2*, *CA3* and *CA4*: CA Sommer’s sectors of the hippocampus; *GD*: dentate gyrus.

**Figure 4:**
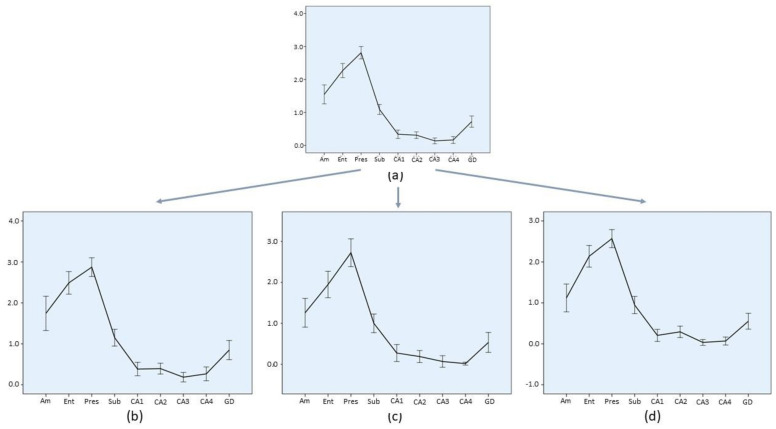
Regional profile of average involvement (Ai) intensity along the medial temporal lobe of sCJD histotype groups. Slight diversity of profiles within the sCJD MM/MV1 subtype is shown. Confidence interval bars (95%) are shown as all groups include n > 30. Note the different scale range of single charts. (**a**) All sCJD MM/MV1 cases, either pure or mixed, as shown in Figure 3a. (**b**) Only pure sCJD MM/MV1 cases with PrP^res^ type 1; (**c**) mixed sCJD MM/MV (1 + 2) cases; and (**d**) pure sCJD MM/MV1 cases excluding cases with atypical phenotype. *Am*: amygdala; *Ent*: entorhinal cortex; *Pres*: presubiculum; *Sub*: subiculum; *CA1*, *CA2*, *CA3* and *CA4*: CA Sommer’s sectors of the hippocampus; *GD*: dentate gyrus.

**Figure 5:**
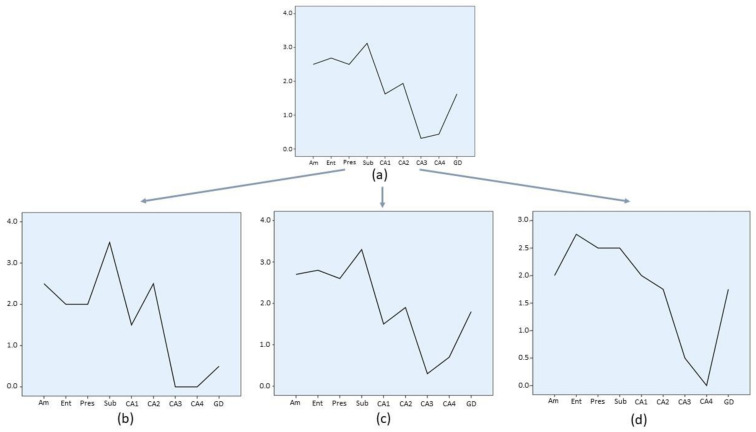
Regional profile of average involvement (Ai) intensity along the medial temporal lobe in sCJD. Range of phenotypic diversity within the sCJD MV2K histotype. Note the different scale range of single charts. (**a**) All sCJD MV2K cases, either pure or mixed type, as shown in Figure 3c; (**b**) pure sCJD MV2K cases; (**c**) cases with a sCJD MV2K+C phenotype; and (**d**) cases showing the sCJD MV2K+1 phenotype. *Am*: amygdala; *Ent*: entorhinal cortex; *Pres*: presubiculum; *Sub*: subiculum; *CA1*, *CA2*, *CA3* and *CA4*: CA Sommer’s sectors of the hippocampus; *GD*: dentate gyrus.

**Figure 6:**
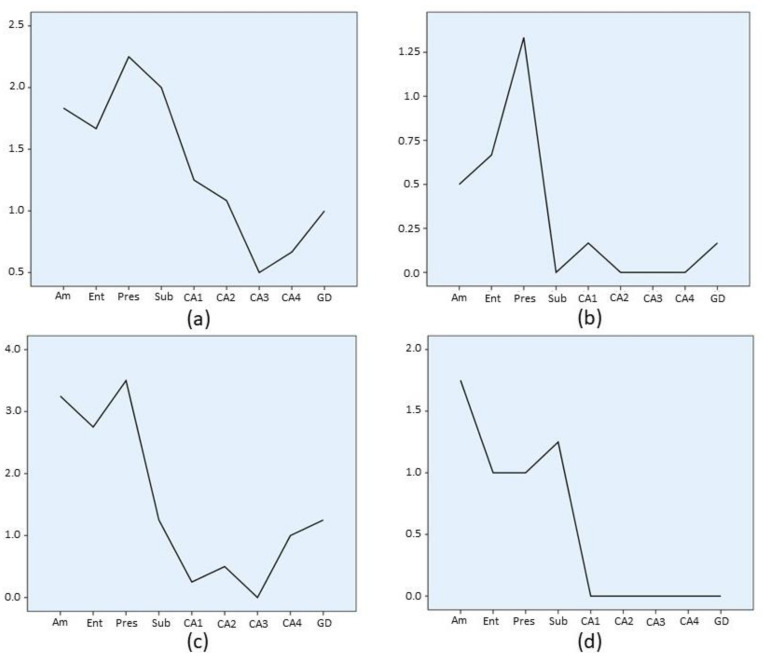
Lesion profile of average involvement (Ai) intensity along the medial temporal lobe in genetic and acquired forms of human prion disease (HPD). Note the different scale range of single charts. (**a**) Familial CJD; (**b**) fatal familial insomnia; (**c**) iatrogenic CJD; and (**d**) variant CJD. *Am*: amygdala; *Ent*: entorhinal cortex; *Pres*: presubiculum; *Sub*: subiculum; *CA1*, *CA2*, *CA3* and *CA4*: CA Sommer’s sectors of the hippocampus; *GD*: dentate gyrus.

In order to summarize regional involvement of MTL structures by HPD, a lesion profile, based on the average involvement score (Ai), was produced for each diagnostic group (Figure 3, Figure 4, Figure 5 and Figure 6). Graphs depicting the lesion profile for each type or subtype of HPD represent the mean Ai for each area of interest. Assessed areas are presented in an anterior to posterior sequence (amygdala → entorhinal cortex and hippocampus) and in a distal to proximal sequence for structures comprising the hippocampal formation (entorhinal cortex → presubiculum → subiculum → CA1 → CA2 → CA3 → CA4 → dentate gyrus). The lesion profiles for sCJD molecular subtypes (Figure 3) represent all cases included in each group, either with a pure or mixed phenotype. Variations of this average profile in different phenotypic combinations vs. pure cases are shown in Figure 4 and Figure 5.

The largest sCJD group, MM/MV1, either pure or mixed (Figure 3a), showed mild to moderate involvement of the amygdala and entorhinal cortex, high grade involvement of presubiculum, followed by a gradient of descending intensity from subiculum to CA4. The slope of decrease was particularly sharp both between the presubiculum and subiculum, and from the subiculum to CA1 sector. In the hippocampal cortex, a minimal degree of involvement was observed in the CA3 sector. Dentate gyrus was invariably, though mildly, affected by pathology. Minimal variations from this common pattern are shown in Figure 4, with slightly less involvement of the proximal hippocampal cortex (CA2 → CA4) in mixed sCJD MM/MV (1 + 2) cases.

In contrast, sCJD VV2 (Figure 3b) cases showed high intensity of involvement from the amygdala to CA2, with a deep decrease of pathology at the CA3 sector and a further sharp increase toward CA4 and dentate gyrus. The pattern displayed by the sCJD MM/MV2C cases (Figure 3c) followed the global profile of MM/MV1 cases, although intensity of CA1 involvement was higher in the former group (Mann–Whitney U test, *p* = 0.66, when comparing the pure cases of both groups). Lesion profile displayed by the sCJD MV2K group (Figure 3d) globally followed the VV2 pattern, with a lower intensity of involvement of the CA1 sector (Mann–Whitney U test, *p* < 0.05). Significant profile variations within the group (Figure 5) were limited to sCJD MV2K + 1 cases, which showed a VV2-like pattern of involvement, with intense pathology at the CA1 sector. Finally, the sCJD VV1 profile should be considered cautiously due to the low number of cases (*n* = 2), though a MM/MV1-like pattern could be observed, with a higher degree of involvement of the amygdala, entorhinal cortex, and dentate gyrus.

Familial CJD cases (E200K mutation) (Figure 6a) showed medium to low intensity involvement with a pattern similar to the sCJD MM/MV2C group. Familial fatal insomnia (Figure 6b) displayed low grade involvement with hippocampal pathology limited to the subiculum. Among the acquired CJD entities, iatrogenic CJD cases (Figure 6c) displayed a pattern similar to sCJD MM/MV1 with a high level pathology at the amygdala, entorhinal cortex, and presubiculum, while variant CJD (Figure 6d) showed no hippocampal cortex pathology (pyramidal layer) with a minimal intensity of lesions in the rest of the assessed areas.

Immunohistochemical patterns of PrP^res^ deposits in the MTL were consistent with those observed in extratemporal CNS regions, although they were not systematically analyzed in this study.

## 4. Discussion

Every neurodegenerative disease displays a characteristic regional distribution of lesions along the central nervous system that results from a twofold process: selective spread of the pathologic protein (Aβ, tau, α-synuclein, TDP-43, PrP^Sc^, among others) along specific connectivity circuits, and selective vulnerability of certain neuron populations to its toxic properties [8]. Disease progression along neuropathological stages (e.g., Braak stages for Alzheimer’s or Parkinson’s disease) represents the recruitment of new populations and circuits by the pathological process. Such a stereotyped spatiotemporal involvement of the nervous system has suggested a comprehension of these entities as “nexopathies” [9]. Posttranslational changes in the pathologic protein, leading to its anomalous conformation, aggregation, and toxicity, are highly determinant of the regional distribution of lesions, and consequently of the resulting neuropathological and clinical phenotype [1,2,3].

Human prion diseases manifest these molecular–neuropathological–clinical correlations in a particularly clear-cut way. For all etiological forms of HPD, the haplotype of *PRNP* gene codon 129 (MV, MM or VV) determines both disease risk and phenotypic diversity [27]. The other major factor determining disease phenotype is PrP^res^ type (e.g., types 1 and 2a for sCJD), as may be obtained through western blot. Both these molecular features are not independent, so that in sCJD type 1, PrP^res^ is predominantly associated with the MM genotype, while type 2a PrP^res^ is associated mainly with VV or MV genotypes at codon 129. While this is the basis for a neat molecular classification of sCJD, genetic and acquired forms of HPD also manifest some more variable dependence of neuropathological and clinical phenotype on the patient’s genotype and PrP^res^ type [7,21].

Original descriptions of sCJD global neuropathological phenotypes [19,28] have already evidenced the existence of prion disease subtypes with specific hippocampal involvement, associated with high burden of pathology in other limbic brain regions (e.g., sCJD VV2 and MV2K). Some studies have suggested a lower general vulnerability of the hippocampus to prion pathology [29,30]. Other researchers have added further detail to the pathological involvement of the medial temporal lobe in particular types and subtypes of HPD [31,32]. Noteworthy, the presence of Hirano body clusters in the CA1 sector has been described in sCJD histotypes VV1 and MV2K [31]. Although not systematically explored in our study, this finding could be documented in some MV2K and panencephalopathic sCJD cases. Here, we present evidence of definite differential patterns of disease involvement of the MTL for different HPD types and subtypes. These patterns are based on the regional distribution of spongiosis and astrogliosis, and not on the presence or intensity of PrP^res^ deposits. According to our results, only FFI and vCJD displayed low grade involvement at all assessed levels. A second group of HPD (fCJD, iCJD, and sCJD MM/MV1, MM/MV2C, and VV1, either pure or mixed) showed medium to high involvement of extra-hippocampal structures with minimal pathology at the hippocampus proper. Finally, a third group of entities including sCJD VV2 and MV2K (either pure or mixed) displayed high level involvement of all MTL structures. For most diagnostic groups, the area showing the highest intensity of pathology was the presubiculum, while minimal involvement was usually limited to the CA3 sector of the hippocampus. A gradient of pathology intensity can commonly be observed between these two neuroanatomical regions.

Different lesion profiles at the MTL may represent the pathogenic effect of specific PrP^Sc^ strains. There is consistent evidence, based on structural biochemical and transmission studies, on the presence of five different PrP^Sc^ strains in sCJD [7]. The M1 strain has been identified in sCJD MM/MV1, iCJD MM1, and several fCJD mutations (E200K, among others) associated with the MM genotype. In the present series, sCJD MM/MV1 cases displayed a rather stable lesion profile that was only minimally modified in mixed type 1 + 2 cases or in panencephalopathic phenotypes. A comparable pattern was observed in our iCJD MM cases. However, fCJD cases (E200K mutation) displayed a lesion profile closer to the sCJD MM/MV2C than to the sCJD MM/MV1 group, though the differences were here slight and limited to CA1 (not significant). Among sCJD histotypes, strain V2 has been identified in VV2 and MV2K cases. Although our results yielded a lesion profile clearly differentiated from sCJD MM/MV1 for both sCJD VV2 and MV2K, these latter groups displayed some specific differences, the main one, once again, at the CA1 sector. Notably, the profile observed in sCJD MV2K + 1 cases was fully comparable to sCJD VV2. The M2T strain was observed in sCJD MM2T and in FFI (D178N-M) cases. No sCJD MM2T cases were included in this series, but our FFI patients displayed quite a sharply differentiated profile with low degree MTL involvement and virtually no hippocampal pathology. As for the M2C strain, detected in sCJD MM/MV2C cases, its main differential feature with sCJDMM/MV1, as previously discussed, was a higher involvement of CA1 (*p* = 0.66). Finally, the V1 strain was found limited to sCJDVV1 cases. Our sCJDVV1 group, although small (*n* = 2), yielded a quite definite profile with very high intensity of involvement of extrahippocampal structures and no hippocampal involvement. The other single strain represented in this series was the BSE agent, characteristic of vCJD cases. The pattern obtained in our study shares with sCJD VV1 a total absence of hippocampal cortex involvement, though a unique feature of this lesion profile is the absence of dentate gyrus involvement. This low hippocampal involvement in vCJD cases has been previously documented [32,33].

**Figure 7:**
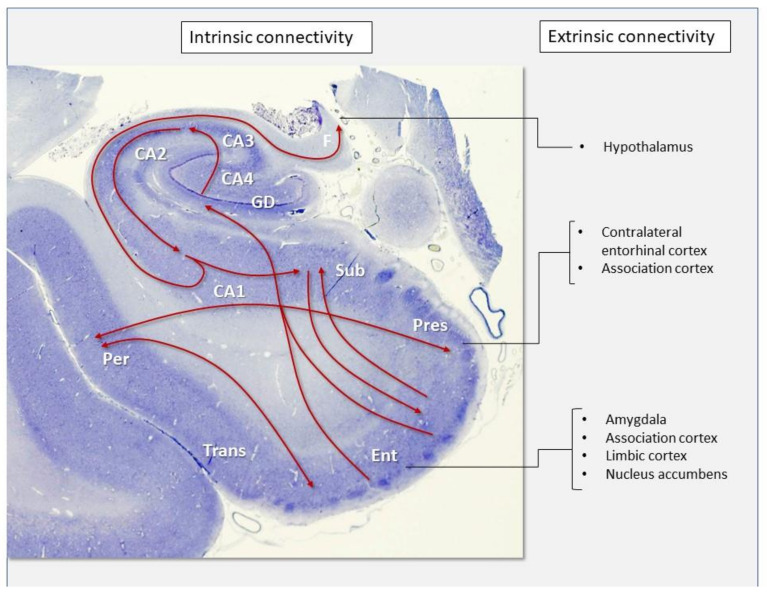
Macroscopic image of the hippocampal formation from a normal control subject, coronal section, Nissl stain. The main pathways of intrinsic and extrinsic connectivity are depicted. *Per*, perirhinal cortex. *Trans*, transentorhinal cortex. *Ent*, entorhinal cortex. *Pres*, presubiculum. *Sub*, subiculum. *CA1*, *CA2*, *CA3* and *CA4*, Sommer’s sectors of the hippocampus cortex. *GD*, dentate gyrus.

Structures within the MTL are deeply interconnected (intrinsic connectivity) and are also widely connected to other cortical and subcortical brain regions (extrinsic connectivity) (Figure 7). The basolateral division of the amygdala receives direct afferent fibers from multiple limbic cortical areas, and projects to these same areas as well as to the hippocampus, dorsomedial thalamic nucleus, and nucleus basalis of Meynert. The entorhinal cortex is the principal afferent pathway to the hippocampus. From the entorhinal cortex, numerous pyramidal cell fibers (layer III) project directly to the dentate gyrus (perforant pathway), while a minor pathway (alvear) projects from layer V to the subiculum and superficial layers of CA1 and CA3. Granular cells of dentate gyrus (mossy fibers) project to CA3, where pyramidal cells in turn project to CA1 through collateral branches (Shaffer’s collaterals) of their axons, which enter the fornix toward the hypothalamus. CA1 is connected to the subiculum, and from this region, fibers are sent back to the entorhinal cortex. The resultant trisynaptic loop is the neuroanatomical basis of short-term memory. Other neural pathways connect profusely diverse hippocampal structures to striatal (nucleus accumbens), septal, hypothalamic, thalamic, and brainstem nuclei [10].

With such a dense extrinsic and intrinsic connectivity of MTL structures, it is not surprising that this brain region is so affected in HPD. However, as the diversity of the here presented lesion profiles indicate, some areas are highly vulnerable to disease while other structures seem to be highly resistant, especially in some disease types or subtypes. Our results show that highly connected nuclei of the amygdala in its basolateral division are invariably involved as well as the entorhinal cortex and one of its main projection structures, the dentate gyrus. In contrast, CA3 and CA4 sectors of the hippocampus commonly show minimal or no involvement in all disease groups. Notably, the highest intensity scores were registered in the presubiculum, the most medial part of the periallocortex (which also includes the parasubiculum and the entorhinal cortex). Studies in nonhuman primates and connectional studies have shown the presubiculum to be the main source of contralateral afferents to the entorhinal cortex as well as connected to the temporal (perirhinal), frontal, and parietal cortices [34].

Most focal neurodegenerative pathological entities, some of them recently described, are limited to the MTL. This group encompasses focal pathologies that are fully limited to the MTL, with minimal extratemporal extension, like argyrophilic grain disease (AGD) and limbic-predominant age-related TDP-43 encephalopathy (LATE), together with focal variants of diffuse diseases. The latter group includes primary aging-related tauopathy (PART) and Lewy body disease, amygdala predominant (LBD-A). All of them are associated with advanced age and appear commonly in combination with other diffuse neurodegenerative disease. Aging-related tau astrogliopathy (ARTAG) will not be here discussed due to its primary glial involvement, which suggests different spreading mechanisms [17]. A brief discussion of these focal neurodegenerative entities is here included, as they may manifest the pathogenic effect of specific protein isoforms that share spreading pathways with already characterized prion strains.

AGD is a 4-repeat (4R) sporadic tauopathy that presents commonly in postmortem studies either in combination with other tauopathies or associated with prevalent neuropathological substrates of dementia in aged patients, namely Alzheimer’s, Lewy body, or cerebrovascular disease [15,35,36,37,38,39]. Rarely, it may be the single or main cause of clinical neurological disease, namely dementia or a neuropsychiatric syndrome [38]. Hence, AGD is currently classified among frontotemporal lobar degenerations, the neuropathological counterpart of frontotemporal dementia. The histological hallmark of AGD is the massive presence of argyrophilic grains, small grain-like 4–8 μm neuronal inclusions containing hyperphosphorylated tau, together with other phospho-tau inclusions characteristic of 4R tauopathies. Early stages of AGD involve the gyrus ambiens and basolateral nuclei of the amygdala (stage I), and pathology extends subsequently to other amygdaloid nuclei, entorhinal cortex, CA1 sector, and temporal neocortex (stage II). Advanced disease is characterized by limited extratemporal extension to the anterior cingulate and insular cortex, nucleus accumbens, septum, and hypothalamus, regions directly connected to the MTL [39]. Regional distribution of AGD pathology displays anterior–posterior, medial–lateral, and distal–proximal gradients of intensity in the MTL and hippocampus as a trace of local propagation along different axes [36]. AGD phospho-tau has been shown to present prion and strain-like properties in experimental transmission to transgenic animals [2,40].

LBD-A is a form of Lewy body disease that appears commonly, though not exclusively, in combination with advanced Alzheimer’s disease, with a higher prevalence among patients over 80 years old (around 5–10% of cases with advanced AD pathology) [14]. It has been considered either as a specific form of LBD or, more recently, as an early stage of rostro-caudal propagation of LB pathology that may start at the olfactory bulb or the amygdala [12,41]. LBD-A shows high density of LB pathology in the amygdala that in most cases extends to the entorhinal, perirhinal, and hippocampal cortices. A higher intensity of pathology has been described in the central nucleus of the amygdala [42]. LB pathology displays specific features in the amygdala in all types of LBD involving this neuroanatomical region, associated with the presence of specific alpha-synuclein isoforms at this level [43].

Like LBD-A, PART has also been interpreted as an early focal stage of a diffuse disease. In this case, however, the diffuse condition, tangle-only dementia, is highly rarer than its focal counterpart. PART is currently classified among 3R+4R sporadic tauopathies and presents Alzheimer’s type neurofibrillary lesions limited to the MTL that are not associated with Aβ pathology of the corresponding intensity [16]. Despite a high similarity to AD pattern of involvement of the MTL, PART displays some specific features (e.g., an earlier and higher involvement of the CA2 sector of the hippocampal cortex) [44,45].

The description of the key role of TDP-43 in amyotrophic lateral sclerosis and frontotemporal dementia has led to research on immunoreactive pathology in hippocampal sclerosis and Alzheimer’s disease [46]. Recently, LATE has been described as a focal TDP-43 pathology with predominant MTL involvement, highly associated with Alzheimer’s disease and other neuropathological substrates of dementia. In the progression of LATE, hippocampal sclerosis, involving subiculum, CA1, and CA2–3 sectors, may develop at an advanced stage of disease. A staging system has been proposed for LATE, which includes an early stage limited to the amygdala (Stage I), an intermediate stage involving the hippocampus (Stage II), and an advanced stage with TDP-43 inclusions present in the frontal cortex (Stage III) [18]. Inclusions in the amygdala are most frequent in the basolateral nuclei and gyrus ambiens, while in the hippocampus, inclusions involve the dentate gyrus, subiculum, and CA sectors, particularly in the presence of hippocampal sclerosis. Evidence is starting to emerge of the existence of conformational variants (pathological conformers) that may explain phenotypic diversity in frontotemporal dementia with TDP-43 inclusions [47]. It will be interesting to know if further research discloses whether LATE is associated with specific strain-like features of the TDP-43 protein.

All the above-mentioned focal neurodegenerative conditions most likely share pathogenic mechanisms for the generation and spread of pathological protein isoforms, with a key role of the amygdala, as has recently been suggested [48]. In contrast, involvement of the MTL in HPD is driven by the rich external connectivity of this brain region. However, our results suggest that once a PrP^res^ strain enters the MTL, it induces a highly stereotyped pattern of involvement, related to the internal connectivity of the region and to selective vulnerability of specific neuronal populations to that particular strain. It is foreseeable that in the near future, research on the structural and transmission properties of focal MTL pathology will reveal specific strains or strain-like features of involved isoforms of tau, α-synuclein, or TDP-43.

## 5. Conclusions

The analysis of the lesion profiles induced in the medial temporal lobe by various types and subtypes of human prion diseases is consistent with the presence of several well-characterized PrP^res^ strains. Different patterns of MTL involvement express the participation of specific connectivity circuits and neuron populations in the pathogenic process. Altogether, these lesion profiles offer a reference framework for the analysis of strain-like phenotypic patterns in non-prion neurodegenerative diseases, and particularly in focal conditions that involve the MTL. Among the latter, AGD and LBD-A, together with recently described entities like PART and LATE, displayed specific patterns of MTL involvement by various pathologic proteins (tau, α-synuclein, and TDP-43). Evidence is rapidly emerging on the strain-like behavior of specific isoforms of these proteins, both in diffuse disease forms and in focal pathologies limited to the MTL.

## Figures and Tables

**Table 1:** Distribution of human prion disease cases between diagnostic entities including some basic sociodemographic and clinical data. Pure and mixed sCJD cases are classified in separate groups.

Disease Type	Subtype	n	Sex (% Female)	Age at Death ^1^	Dementia at Onset (%)
Sporadic CJD	MM/MV1	56	46.4	67.5 (10.1)	52.9
(pure)	VV2	20	60	69.2 (7.2)	50
	MV2K	2	50	67.5 (9.2)	50
	MM/MV2C	4	50	66.3 (4.7)	50
	VV1	2	0	46 (13)	50
Sporadic CJD	MM/MV (1 + 2)	31	51.6	69.1 (1.6)	53.3
(mixed)	MM/MV2C + 1	2	50	70 (7.1)	100
	MV2K + 1	3	66.7	70.3 (8)	66.6
	MV2K + C	6	66.7	67.7 (14.2)	40
Familial CJD		8	25	61.1 (7.9)	37.5
FFI ^2^		4	50	52.5 (13)	33.3
iCJD ^3^		2	50	41.5 (6.4)	0
Variant CJD		3	33.3	39 (12.6)	33.3

^1^ Mean (SD). ^2^ Familial fatal insomnia. ^3^ Iatrogenic CJD.

**Table 2:** Average involvement score (Ai, mean, [SD]) for all assessed structures of the medial temporal lobe in different types and subtypes of human prion diseases included in the patient series.

Disease Type	Subtype	Amy	Ent	Pres	Sub	CA1	CA2	CA3	CA4	DG
sCJD	MM/MV1	1.71 (1.3)	2.39 (0.88)	2.82 (0.8)	1.15 (0.65)	0.39 (0.55)	0.41 (0.47)	0.16 (0.35)	0.25 (0.52)	0.84 (0.75)
(pure)	VV2	2.75 (0.43)	2.86 (0.45)	3.03 (0.65)	2.92 (0.55)	2.81 (0.42)	2.47 (0.58)	1.14 (1.01)	1.72 (0.75)	2.06 (0.99)
	MV2K	-	2.25 (0.35	2.5 (0.71)	3.25 (0.35)	2.25 (1.06)	2.75 (0.35)	0.25 (0.35)	0.5 (0.71)	1 (0.71)
	MM/MV2C	1.25 (0.5)	2.38 (0.48)	2.5 (0.41)	1.13 (0.48)	0.88 (0.75)	0.38 (0.25)	-	0.13 (0.25)	0.63 (0.25)
	VV1	3 (1.41)	2.5 (2.12)	-	2.75 (1.77)	1 (0.71)	1 (0.71)	1 (0.71)	0.75 (1.06)	2.25 (1.06)
sCJD	MM/MV (1 + 2)	1.3 (0.93)	1.99 (0.83)	2.73 (0.88)	0.97 (0.61)	0.27 (0.54)	0.18 (0.38)	0.67 (0.36)	0.17 (0,09)	0.52 (0.64)
(mixed)	MM/MV2C + 1	1.5 (1.41)	-	-	-	-	-	-	-	-
	MV2K + 1	2 (0.71)	2.5 (1.32)	2.5 (1.41)	2 (1.32)	1.67 (1.61)	1.5 (1.8)	0.33 (0.58)	-	1.33 (1.04)
	MV2K + C	2.33 (1.21)	2.67 (0.52)	2.17 (1.29)	3.17 (0.41)	1.25 (1.37)	1.58 (1.28)	0.3 (0.45)	0.7 (0.45)	1.8 (0.84)
Familial CJD		1.71 (1.07)	1.83 (1.25)	2.36 (0.89)	1.86 (0.89	1.21 (0.99)	1 (1)	0.43 (0.61)	0.64 (0.89)	1.14 (1.14)
FFI ^1^		0.63 (0.48)	0.67 (0.29)	1.5 (0.58)	0.25 (0.5)	0.16 (0.25)	-	-	-	0.38 (0.48)
iCJD ^2^		3.25 (0.75)	2.75 (1.06)	3.5 (0.71)	1.25 (0.35)	0.25 (0.35)	-	-	1 (1.41)	1.25 (1.06)
Variant CJD		1.75 (1.77)	1 (1.41)	1.33 (1.15)	1.33 (1.26)	-	-	-	-	0.33 (0.58)

^1^ Familial fatal insomnia. ^2^ Iatrogenic CJD.

**Table 3:** Vacuolation pattern observed in assessed areas of the medial temporal lobe in different types and subtypes of human prion diseases included in the patient series. The predominant pattern is shown (code number), followed by less frequent patterns in decreasing order ^1^. Numbers separated by “;” represent patterns equally represented in each area.

Disease Type	Subtype	Amy	Ent	Pres	Sub	CA1	CA2	CA3	CA4	DG
sCJD	MM/MV1	1 > 5 > 2	1 > 5 > 2	1 > 5 > 2	1 > 5 > 2	5 > 1	1 > 5	5 > 1	5 > 1	1 > 5 > 2
(pure)	VV2	2 > 1	2 > 1	2 > 1 > 5	2 > 1	2 > 1	2 > 1	2 > 5 > 1	2 > 1 > 5	2 > 1 > 5
	MV2K	2	1; 2	1; 2	1; 2	1; 2	1; 2	5	1; 5	1
	MM/MV2C	3 > 1; 4	3 > 4	3 > 1	3 > 1	3	3	5	5 > 1	3 > 1
	VV1	1; 5	1; 5	5	1; 5	1; 5	1; 5	1; 5	1; 5	1; 2
sCJD	MM/MV (1 + 2)	1 > 5 > 3 > 4 > 2	1 > 3; 4 > 2; 5	1 > 5 > 3 > 4 > 2	1 > 3; 5 > 2; 4	5 > 1 > 2; 4	5 > 1 > 4	5	5 > 1	1 > 5 > 2; 3
(mixed)	MM/MV2C + 1	1; 3	2	-	3	5	5	5	5	5
	MV2K + 1	2	1; 2; 3	1; 2	1; 2; 3	1; 2; 3	2; 3; 5	5 > 2	5	2 > 3
	MV2K + C	2 > 1	2	2 > 1; 5	2	2 > 5	2 > 1; 5	5 > 2	2 > 5	2 > 1; 4
Familial CJD		1 > 2; 5	1 > 2 > 5	1 > 2; 5	1 > 2	1 > 2; 5	3 > 2; 5	5 > 2 > 1	5 > 2 > 1	1 > 2; 5
FFI ^1^		1 > 5	1	1	5 > 1	5 > 1	5	5	5	1; 5
iCJD ^2^		1; 5	1; 5	1; 5	1	1; 5	1	5	1; 5	1
Variant CJD		2	2; 5	2 > 5	2 > 5	5	5	5	5	5 > 2

Note: 1 Only small vacuoles; 2 only medium size-large vacuoles; 3 predominantly small vacuoles; 4 predominantly medium size-large vacuoles; and 5 minimal vacuolation. ^1^ Familial fatal insomnia. ^2^ Iatrogenic CJD.

## Data Availability

The data presented in this study are available on request from the corresponding author. The data are not publicly available due to ethical reasons.
